# A novel understanding of postoperative complications: *In vitro* study of the impact of propofol on epigenetic modifications in cholinergic genes

**DOI:** 10.1371/journal.pone.0217269

**Published:** 2019-05-29

**Authors:** Caroline Holtkamp, Björn Koos, Matthias Unterberg, Tim Rahmel, Lars Bergmann, Zainab Bazzi, Maha Bazzi, Hassan Bukhari, Michael Adamzik, Katharina Rump

**Affiliations:** 1 Klinik für Anästhesiologie, Intensivmedizin und Schmerztherapie, Universitätsklinikum Knappschaftskrankenhaus Bochum-Langendreer, Ruhr-Universität Bochum, Bochum, Germany; 2 Medizinisches Proteomcenter (MPC), Ruhr-Universität Bochum, Bochum, Germany; Weizmann Institute of Science, ISRAEL

## Abstract

**Background:**

Propofol is a widely used anaesthetic drug with advantageous operating conditions and recovery profile. However, propofol could have long term effects on neuronal cells and is associated with post-operative delirium (POD). In this context, one of the contributing factors to the pathogenesis of POD is a reduction of cholinesterase activity. Accordingly, we investigated the effects of propofol on the methylation, expression and activity of cholinergic genes and proteins in an *in-vitro* model.

**Results:**

We found that propofol indeed reduced the activity of AChE / BChE in our *in-vitro* model, without affecting the protein levels. Furthermore, we could show that propofol reduced the methylation of a repressor region of the *CHRNA7* gene without changing the secretion of pro–or anti-inflammatory cytokines. Lastly, propofol changed the expression patterns of genes responsible for maintaining the epigenetic status of the cell and accordingly reduced the tri-methylation of H3 K27.

**Conclusion:**

In conclusion we found a possible functional link between propofol treatment and POD, due to a reduced cholinergic activity. In addition to this, propofol changed the expression of different maintenance genes of the epigenome that also affected histone methylation. Thus, propofol treatment may also induce strong, long lasting changes in the brain by potentially altering the epigenetic landscape.

## Introduction

Propofol is a widely used short-acting intravenous anaesthetic drug with advantageous operating conditions and recovery profile [1, 2]. However, there are several indications that anaesthetics might induce unwanted long-lasting side effects, affecting the central nervous system and cognitive abilities [3–5]. Potentially, this comes to pass by changing the epigenetic profile of the cells [6, 7], which could cause post-operative complications. One of these cerebral complications after surgery is postoperative delirium (POD), which is statistically associated with propofol anaesthesia [8, 9]. POD depicts an acute brain failure [10], which occurs in 15–53% of older patients after surgery and anaesthesia [11, 12] and is associated with an adverse outcome [13]. In addition, delirium is also linked to an increased risk of long term cognitive defects that recover with high inter-individual differences from days to months [14]. Currently, a pathogenesis, involving a reduced cholinergic activity [15], neuroinflammation [16, 17] or a decreased antiinflammation, is discussed in the field [18]. How and if propofol might influence these factors is currently unknown. However, it seems appropriate to speculate that one possible mechanism is an alteration of the epigenetic profile of the cells. Especially the methylation of the promoter regions of the cholinergic genes *ACHE* (acetylcholinesterase), *BCHE* (buturylcholinesterase) and *CHRNA7 (*the subunit alpha 7 of the nicotinic acetylcholine receptor) are of importance. The protein AChE is the primary acetylcholineesterase, hydrolysing acetylcholine at an enormous rate into acetic acid and choline [19]. BChE on the other hand is an unspecific cholinesterase, hydrolysing a range of different choline esters. The activities of both, AChE and BChE, are associated with POD [20, 21]. In addition, *CHRNA7* is coding for an ion channel receptor capable of binding acetylcholine mediating acetylcholine signalling. It has special significance for higher cognitive functions and is linked to Alzheimer’s Disease’s progression [22]. Furthermore, Chrna7 is linked to the so called cholinergic anti-inflammatory pathway [23].

Therefore, in this study we investigated whether the expression, activity and methylation profile of cholinergic genes are changed by propofol. Moreover, we studied how propofol changes the global epigenetic landscape of the cell. And we also investigated the role of *CHRNA7* as mediator between cholinergic proteins and cytokine release.

## Materials and methods

### Cell culture

The human neuroblastoma cells SH-SY5Y (origin: Cell Lines Service, CLS, Eppelheim Germany, SH-SY5Y item number: 300154) were cultured at 37°C and 5% CO_2_ in Dulbecco’s modified Eagle medium (DMEM; Gibco, Darmstadt, Germany) with 10% foetal calf serum (FCS; Gibco, Darmstadt, Germany) and 1% penicillin/streptomycin (Penstrep; Gibco, Darmstadt, Germany). Cells were split twice a week by aspirating medium and the addition of 5 ml Trypsin-EDTA 2.5% (Gibco, Darmstadt, Germany) to dissolve adhesive cells. In addition, peripheral blood mononuclear cells (PBMCs) were studied, after the Ethics Committee’s approval (Ethics Committee of the Ruhr-University Bochum, Bochum, Germany; ref: 17-5964-BR), registration at the German Clinical Trials Register (ref: DRKS00012961, https://www.drks.de/drks_web/navigate.do?navigationId=trial.HTML&TRIAL_ID=DRKS00012961) and written informed consent. An amount of 80 ml EDTA blood from eight healthy donors was taken and PBMCs were isolated, using Ficoll-Paque (GE Healthcare, Chalfont, UK).

### Quantitative reverse transcription PCR

qRT-PCR on SH-SY5Y cells and PBMCs was done as described previously [24]. Briefly, cells were seeded in 6-well culture dishes and stimulated with 25 μg/ml propofol for 2, 4 and 24 h or were left unstimulated (control). Cells were incubated at 37°C and 5% CO_2_. After RNA isolation and cDNA synthesis of 1 μg RNA using the QuantiTect Reverse Transcription kit (Qiagen, Hilden, Germany), we subjected 2 μl of cDNA together with specific primers (Table 1) and GoTaq qPCR master mix (Promega, Madison, WI, USA) to a standard qPCR reaction protocol.

**Table 1:** Primer pairs for quantitative real-time PCR.

Primer name	Sequence (5’ to 3’)	Product size (bp)
ACHE_M5_SE	TTTTTAATTAGTGCGGTTAGAACGT	145
ACHE_M5_AS	AATATTAAAAAAATAAACCCCTCGC	
ACHE_U5_SE	TTTTTAATTAGTGTGGTTAGAATGT	144
ACHE_U5_AS	ATATTAAAAAAATAAACCCCTCACC	
CHRNA_M2_SE	TTTTGGAGTTTTAAAAGAATTTCGT	174
CHRNA_M2_AS	TCCCTTCTACTAAACACAACAACG	
CHRNA_U2_SE	TTTTGGAGTTTTAAAAGAATTTTGT	174
CHRNA_U2_AS	TCCCTTCTACTAAACACAACAACAC	
BCHE_M1_SE	ATTTAGGTTAAAACGGTGAAATTTC	172
BCHE_M1_AS	AAACTAAAATACCGTAACGCGAT	
BCHE_U1_SE	TTAGGTTAAAATGGTGAAATTTTGG	173
BCHE_U1_AS	CTCAAACTAAAATACCATAACACAAT	
ACTB_SE	CTGGAACGGTGAAGGTGACA	140
ACTB_AS	AAGGGACTTCCTGTAACAATGCA	
DNMT1_RT1_SE	CTGAGGCCTTCACGTTCA	274
DNMT1_RT1_AS	CTCGCTGGAGTGGACTTGT	
DNMT3B_RT3_SE	AATGTGAATCCAGCCAGGAAAGGC	191
DNMT3B_RT3_AS	ACTGGATTACACTCCAGGAACCGT	
KDM2A_SE	CTTTTCCTGGTCGCTCTGAC	237
KDM2A_AS	TCGGGTTCCATCTCTCACTCT	
ACHE_mRNA_SE	GCT TCA GCA AAG ACA ACG AG	115
ACHE_mRNA_AS	GTG TAA TGC AGG ACC ACA GC	
CHRNA7_mRNA_SE	TTT ACA GTG GAA TGT GTC AGA ATA TCC	125
CHRNA7_mRNA_AS	TGT GGA ATG TGG CGT CAA G	
BCHE_mRNA_SE	ATCCTGCATTTCCCCGAAGT	239
BCHE_mRNA_AS	CCGTGCCACCAAAAACTGTC	
ACHE_Prom_SE	GTATTGCCGCATGCACCTC	225
ACHE_Prom_AS	TTCGGACTTTCGTCACCAGG	
CHRNA7_Prom_SE	ACACATTGGCGGCATCTCTC	103
CHRNA7_Prom_AS	TTTGCTTTCCGCACCGTTTG	
BCHE_Prom3_SE	GCATGTGCACTGCAAGTTGA	123
BCHE_Prom3_AS	CCCTGCAGGCAGTCATACAT	
HDAC1_mRNA_AS	TGGCCTCATAGGACTCGTCA	231
HDAC1_mRNA_SE	TGCTAAAGTATCACCAGAGGGT	
ACHE-S_SE	GCGACCACAATGTCGTGT	496
ACHE-S_AS	TTCCAGTGCACCATGTAGGA	
ACHE-S_Probe	FAM-GGGGCTCAGCAGTACGTTAG-TAMRA	
ACHE-Isoform_4_SE	GGCCTGCAGCTGGCT	145
ACHE-Isoform_4_AS	GGGGATCCCAAAGATGAACT	
ACHE-Isoform_4_Probe	FAM-TACGTCTTTGAACACCGTGC-TAMRA	
ACHE-E_SE	GGGGCTCAGCAGTACGTTAG	459
ACHE-E_AS	TGGCTTTTCCATTTCCATTC	
ACHE-E-Probe	FAM-CGCCACCGCCTCGGA-TAMRA	

### Cholinesterase activity and concentration assays after stimulation

Cholinesterase activity, acetylcholine esterase (AChE) and butyrylcholine esterase (BChE) concentration in SH-SY5Y cells were measured after stimulation with propofol or the proinflammatory cytokine tumour necrosis factor alpha (TNFα).

For this purpose, 5 x 10^5^ cells of SH-SY5Y were plated out in 4 ml growth medium containing 10% FBS. Cells were incubated for 24 h at 37°C and stimulated for 2, 4 and 24 h with either 25 or 30 μg/ml propofol (dissolved in ethanol, Sigma-Aldrich, Taufkirchen, Germany) or 10 ng/ml TNFα (dissolved in PBS; Pepro Tech, New Jersey, USA) or were left unstimulated (control with PBS and ethanol).

In the next step, the supernatants were collected and the proteins were isolated from cells: The medium was aspirated, 5 ml of PBS was added, the cells were mechanically dissolved from the wells, transferred into a 1.5-ml reaction tube and centrifuged for 5 min at 13000 x g. Cell pellets were resuspended in 100 μl PBS and sonicated on ice. After centrifugation for 5 min at 5000 x g and 4°C, the supernatant was snap-frozen and stored at -80°C for further analysis.

Protein quantification was performed using the Rotiquant universal kit (Roth, Karlsruhe, Germany), following the manufacturer’s instructions.

The cell lysats and supernatants were used to detect the cholinesterase activity, AChE and BChE concentration. For this the acetylcholinesterase assay kit (fluorometric-red) (abcam, Cambridge, UK), the human AChE ELISA Kit and the BChE ELISA Kit (both Elabscience, Houston, TX, USA) were utilised, according to the manufacturer’s instructions.

### Methylation and expression of cholinergic genes after stimulation

The DNA promoter methylation of cholinergic genes in neuronal cells was quantified using methylation-specific PCR after bisulphite conversion, before and after stimulation. For this purpose, 5 x 10^5^ cells of SH-SY5Y cells were seeded per 4 ml in 6-well culture dishes and incubated for 24 h at 37°C and 5% CO_2_. The cells were stimulated with 25 μg/ml propofol for 2, 4 and 24h. Afterwards, the DNA was isolated after washing with ice-cold PBS using the QIAamp DNA blood mini kit (Qiagen, Hilden, Germany), following the manufacturer’s instructions. The EZ DNA methylation-gold kit (Zymo Research, Irvine, CA, USA) was used for bisulphite conversion. All samples were diluted to 10 ng DNA/μl. Real-time PCR was executed to detect methylation, as described previously [20], utilising the GoTaq qPCR master mix (Promega, Madison, WI, USA) and specific primers (Table 1).

The percentage of methylation was analysed as previously [25, 26][25, 26]. In a final step, the methylation of the stimulated and unstimulated cells was compared.

### Splice variant specific expression of *ACHE-S*, *ACHE-E* and *ACHE-Isoform 4*

In order to study the expression of the three different major splice variants of *ACHE* we performed a quantitative RT PCR based on dual labelled probes. Briefly, after RNA isolation and cDNA synthesis of 1 μg RNA using the QuantiTect Reverse Transcription kit (Qiagen, Hilden, Germany), we subjected 2 μl of cDNA together with primer and probes specific to *ACHE-S*, *ACHE-E* or *ACHE-Isoform 4* splice variants (Table 1) to a quantitative RT PCR reaction. In addition to these experiments, we also evaluated the expression of *BCHE* after propofol treatment using specific primers (Table 1). The data was analysed by using the delta delta Ct method with beta actin expression as reference gene.

### Methylation and expression after ADC incubation

Additionally, the methylation and expression of cholinergic genes after incubation with 50 μM 5-Aza-2’-deoxycytidin (ADC, Sigma-Aldrich, Taufkirchen, Germany) for 72 h was measured. Cells were lysed after ADC incubation. The DNA was extracted, and the methylation was quantified as described above. The RNA was extracted for expression analysis using the RNeasy MiniKit (Qiagen, Hilden, Germany), following the manufacturer’s instructions. An amount of 1 μg RNA was utilised to synthesize the cDNA with the QuantiTect Reverse Transcription kit (Qiagen, Hilden, Germany), according to the manufacturer’s instructions. Quantitative RT PCR was performed, as described above [24], using specific primers for the acetylcholinesterase gene (*ACHE)*, *BCHE* gene (*BCHE)*, nicotinic acetylcholine receptor 7 gene (*CHRNA7)* and actin beta gene (*ACTB)* (reference) (Table 1). The data was analysed using the delta delta Ct method.

### Analysis of histone modifications

Besides, histone modifications of histone 3 after stimulation were analysed. The neuronal SH-SY5Y cells were plated out, as described above, and stimulated with either 25 μg/ml propofol, 10 ng/ml TNFα or left unstimulated (control) for 24 h.

Regarding the acid-based histone extraction, medium was aspirated, 5 ml of PBS was added, cells were dissolved, transferred into a 1.5-ml reaction tube, centrifuged for 5 min and the supernatant was discarded. The resulting pellet was resuspended in 100 μl TEB buffer (containing PBS, 0.5% Triton X 100 (NP-40), 2 mM PMSF and 0.02% NaN_3_; Roth, Karlsruhe, Germany) and incubated at 4°C for 10 min while shaking at 14000 rpm and, subsequently, centrifuged at 3000 g and 4°C for 10 min. After acid extraction in 50 μl 0.2 NHCl overnight, histones were collected by centrifugation for 10 min and 4°C.

Histone concentration was determined using the Rotiquant universal kit (Roth, Karlsruhe, Germany) and histone modification was quantified by ELISA using 50 ng protein for the Path Scan Tri-Methyl Histone H3 (Lys27) (Cell Signaling Technology, Cambridge, UK) ELISA kit.

### Chromatin immunoprecipitation assay (ChIP assay)

A ChIP assay was used to analyse whether the promoter of the cholinergic genes *ACHE*, *BCHE* and *CHRNA7* bind to histone 3 lysine 27. For this purpose, 1 x 10^6^ SH-SY5Y cells per well were plated out. After incubation for 24 h, the Pierce agarose Chip kit (Thermo Fisher Scientific, Waltham, MA, USA) was used, following the manufacturer’s instructions. The H3 K27me3 polyclonal antibody (EpiGentek, Farmingdale, NY, USA) was utilized as a specific antibody. As a positive control an antibody against RNA polymerase II in combination with specific primers against GAPDH while Rabbit IgG in combination with our primers against *ACHE*, *BCHE* and *CHRNA7* promoter regions (see below) was used as a negative control. After DNA isolation, PCR (New England Biolabs, Frankfurt am Main, Germany) was carried out with ACHE_prom, BCHE_prom and CHRNA7_prom primers (Table 1), and the PCR products were analysed on agarose gel (Peqlab, Erlangen, Germany).

### Enzyme linked immunosorbent assay (ELISA) for quantification of cytolines (TNFα, IL-6 and IL-10)

In order to measure the cytokine release from SH-SY5Y cells after propofol treatment we used the ELISA kits for quantification of TNFα, IL-6 and IL-10 (product numbers 430208, 430508, 430608 respectively, all BioLegend, San Diego, CA), according to manufacturer’s recommendations. Briefly, cells were treated with 25 μg/mL propofol and left to incubate for 2h, 4h and 24h in complete growth medium. Supernatant of the cells were taken and applied to the ELISA plate. After washing, detection antibodies conjugated to horseradish peroxidase were applied and incubated. After washing away any excess antibodies, we added the HRP substrate and left the reaction for 10 min before stopping it. The resulting colour change was measured at 450 nm.

### Statistics

All experiments were performed in duplicate and repeated at least three times. Results are presented as mean ± standard deviation. If not otherwise stated, all dataset were analysed using a Wilcoxon non parameteric test (for multiple comparisons) with a Man Whitney Test for specific comparisons. A p-value < 0.05 was regarded as statistically significant. For multiple comparisons, specific comparisons were only analysed if the Wilcoxon test showed a statistically significant difference between the groups. All statistical analyses were performed using SPSS 25 (IBM, CA, USA)

## Results

### Methylation in cholinergic genes after stimulation with propofol

**Fig 1:**
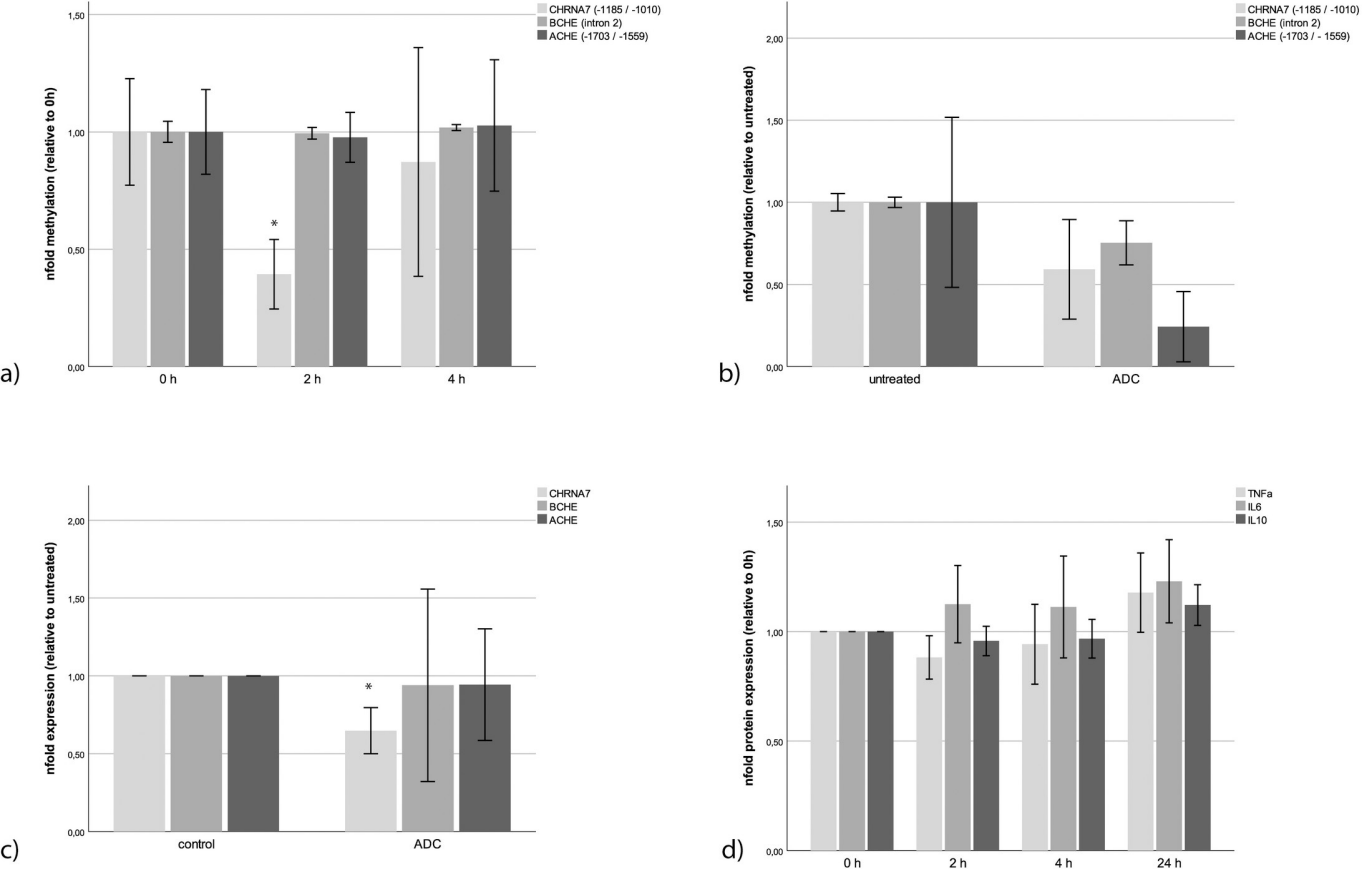
Methylation of cholinergic genes in neuronal SH-SY5Y cells after stimulation (n = 3). (**a**) We could not detect any significantly modified methylation in the promoter of the *ACHE* gene (area -1703/-1559) and *BCHE* gene (intron 2), apart from a decreased methylation in the promoter of the *CHRNA7* gene (area -1185/-1010; p = 0.011) after stimulating SH-SY5Y cells with 25 μg/ml propofol for 2 h. Methylation of *CHRNA7* recovered after 4h to almost unstimulated levels. (**b**) The artificial de-methylation of the epigenome of SH-SY5Y cells using 5-aza-2’ deoxycytidine (ADC, 50 μM) resulted in a visible decrease of overall methylation as measured in the three promoter regions. (**c**) This de-methylation reduced the expression of *CHRNA7* mRNA (p = 0.034) but did not affect *ACHE* or *BCHE* significantly. (**d**) Propofol did not change the expression of proinflammatory (TNFα and IL-6) and anti-inflammatory cytokines (IL-10). Error bars depict 2 x SE.

Propofol stimulation for 2 h decreased the methylation of the promoter region of the *CHRNA7* gene (area nt -1185/-1010) more than 60% compared to the control (p = 0.011; Fig 1A). The methylation of *ACHE* (area nt -1703/-1559) and *BCHE* (intron 2) genes was not affected by propofol (Fig 1A).

### Methylation changes expression of *CHRNA7*

After ADC incubation, the methylation of the *CHRNA7* promoter region (nt -1185/-1010) was decreased by about 30% (Fig 1B) by ADC. While *BCHE* (intron 2) was unaffected, the methylation of the *ACHE* promoter region nt -1703/-1559 was reduced by more than 50% (Fig 1B). However, methylation did not influence *ACHE* or *BCHE* gene expression but decreased *CHRNA7* to roughly 65% residual expression (p = 0.037) (Fig 1C).

### Propofol does not change expression of pro–or anti–inflammatory cytokines

Incubation of the cells with propofol did not significantly change the expression of the cytokines TNFα, IL-6 and IL-10 over 24h. However, we could observe a trend to increased expression of all three cytokines after 24h (p = 0.064 Man Whitney test).

### Cholinesterase activity in SH-SY5Y cells is initially reduced after stimulation with propofol and TNFα

**Fig 2:**
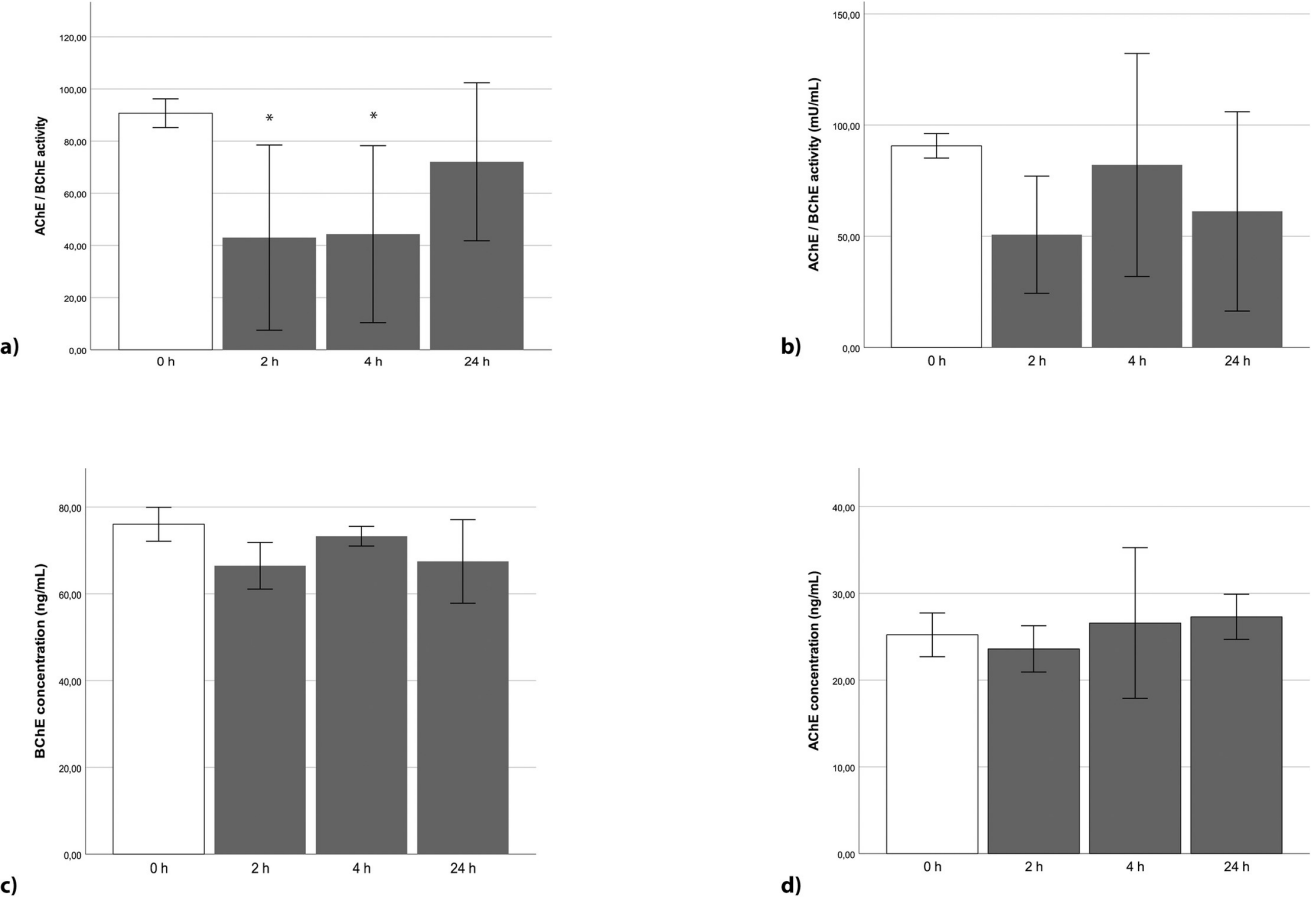
Cholinesterase activity in neuronal SH-SY5Y cells after stimulation (n = 3). (a) After stimulation for 2 h and 4 h with propofol [25 μg/ml], a reduced cholinesterase activity by about 45 mU/ml was measured, compared to the control (p = 0.034 each). Stimulating the cells for 24 h did not present a significant change. (b) TNFα [10 ng/ml] did not significantly reduce cholinesterase activity over the course of 24 h. (c) Propofol stimulation [30 μg/ml] neither did significantly change BChE concentration, (d) nor did it alter the concentration of AChE over the time course of 24h. Error bars depict 2 x SE.

Cholinesterase activity in SH-SY5Y cells was reduced after 2 and 4 h stimulation with propofol to almost 50% in comparison to unstimulated cells (p = 0.034, 2 h; p = 0.034, 4 h) and recovered after 24 h to the initial activity (Fig 2A).

TNFα caused a visible decrease of cholinesterase activity after incubation for 2 h (but did not reach significant levels) in comparison to control values.

The BChE concentration in SH-SY5Y cells was reduced only slightly by 13% after 2 h stimulation with propofol (Fig 2C), but similarly did not reach significant levels. The AChE concentration also was not significantly altered by propofol over the course of the experiment (Fig 2D).

### Splice variant specific expression changes upon propofol treatment

**Fig 3:**
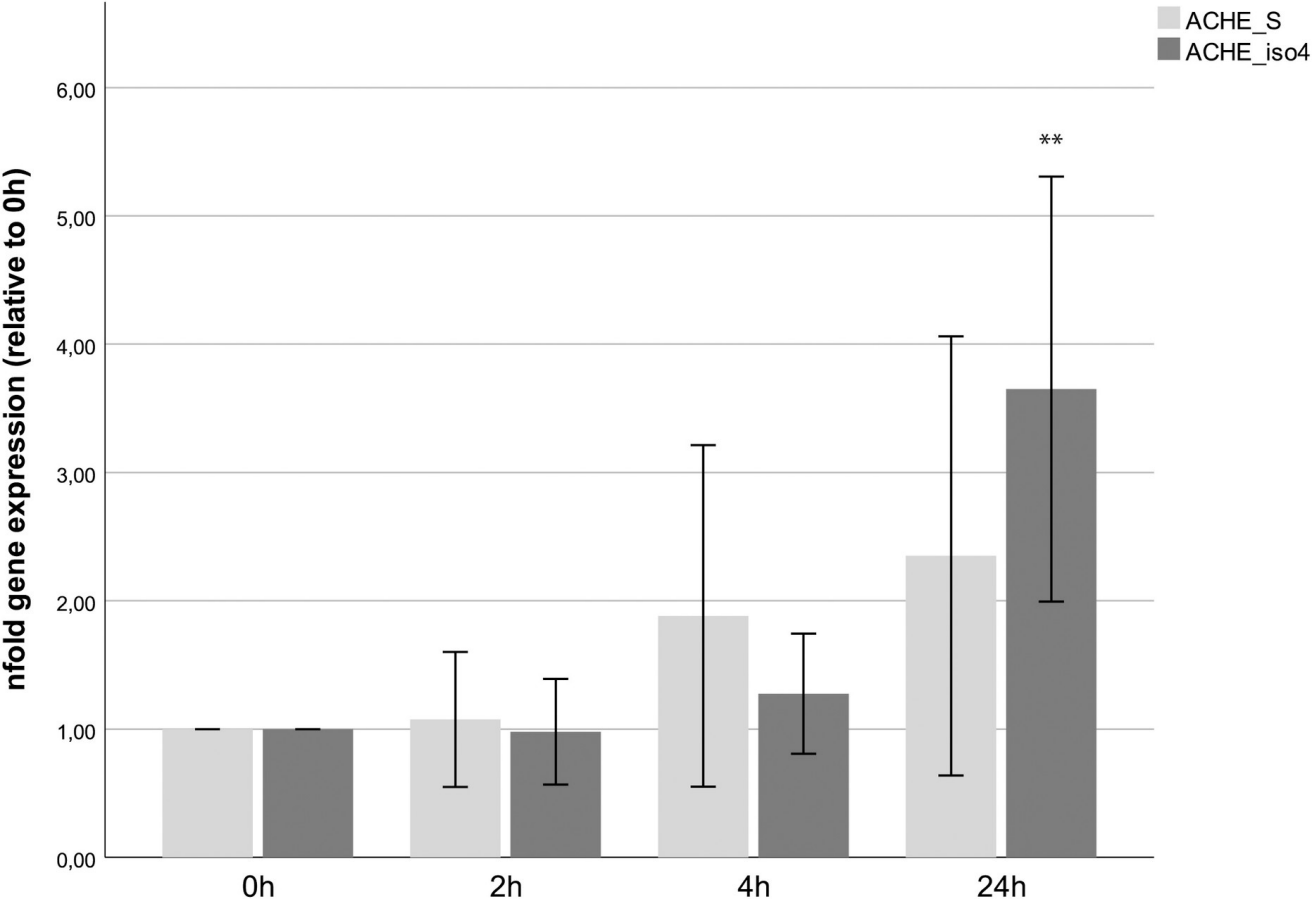
mRNA expression of major splice variants of *ACHE*. Expression of the two major splice variants *ACHE-S* and *ACHE-isoform 4* did not change in the first 4 hours of propofol treatment. Only after 24h the expression of *ACHE-isoform 4* increased significantly (p = 0.005), while the increase observed for *ACHE-S* did not rise to significant levels. Error bars depict 2 x SE.

We tested the expression of all three major splice variants of *ACHE* (*ACHE-S*, *ACHE-E* and *ACHE-isoform 4*) over 24h after propofol stimulation. In our cell line, we could not detect any *ACHE-E* isoform (data not shown), so we concentrated on *ACHE-S* and *ACHE-isoform 4*. Over the course of the first 4 hours we could not find significant expression changes on the mRNA level. Only after 24h of propofol incubation we could see an increase in both *ACHE-S* and *ACHE-isoform 4* expression (to 2.35 fold +/- 1.91 and 3.65 fold +/- 1.85 respectively, Fig 3). Of these only the increase observed for isoform 4 rose to significant levels (p = 0.005) The expression of *BCHE* was not affected by propofol treatment on the mRNA level (data not shown).

### Propofol reduces tri-methylation of histone 3, which binds promoter regions of cholinergic genes

**Fig 4:**
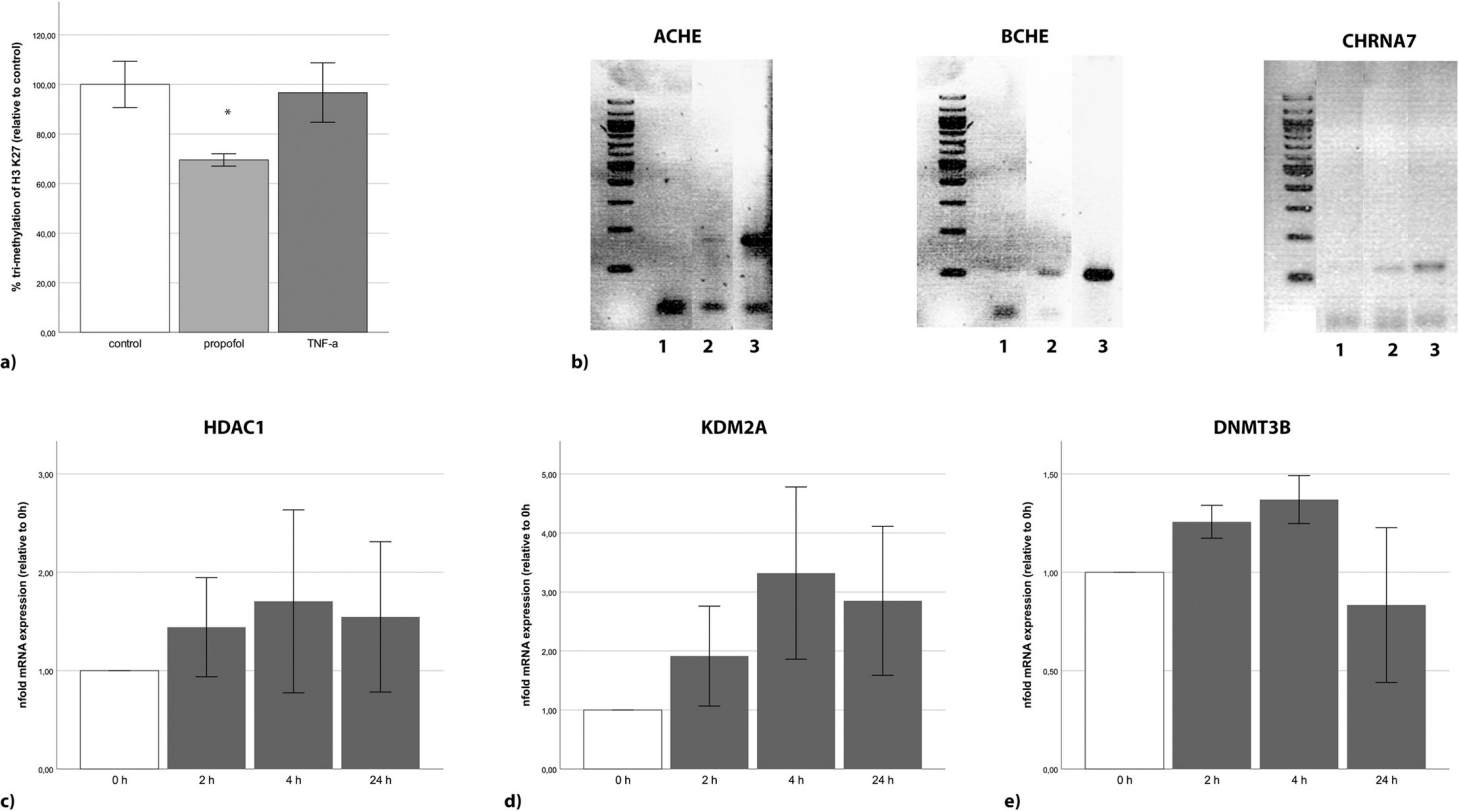
Unspecific changes in the epigenome by propofol. (**a**) The tri-methylation of H3 K27 in SH-SY5Y cells was significantly decreased by propofol [25 μg/ml], while TNFα [10 ng/ml] did not show any changes after 24 h. (**b**) ChIP assays showed that the promoter regions of *ACHE*, *BCHE* and *CHRNA7* bind to tri-methylated H3 K27. For the negative control (1) rabbit IgG was used instead of the specific tri met H3 K27 antibody (2), and the positive control contained an antibody against RNA polymerase and primers for GAPDH. (**c**) Propofol increased the expression of the histone de-acetylating HDAC1 approximately 1.5-fold. (**d**) The expression of KDM2A was increased more than 3-fold after 4h, and (**e**) the expression of DMNT3B was slightly increased over the first 4 hours. Error bars depict 2 x SE.

Propofol reduced the lysine 27 tri-methylation at histone 3 in SH-SY5Y cells by 30% (p = 0.013; Fig 4A). By contrast, TNFα did not affect the methylation of H3K27. The ChIP assay revealed that *ACHE*, *BCHE* and *CHRNA7* promoters all bind to the tri-methylated lysine 27 of histone 3 (Fig 4B). The negative control (rabbit IgG) and the positive control (RNA Polymerase and GAPDH primer) showed the technical feasibility of the data.

### Expression of histone- and DNA-modifying enzymes is associated with propofol incubation

The expression of HDAC1 in SH-SY5Y cells was already increased after 2h of propofol treatment and lasted through 24 h (Fig 4C). Propofol increased the expression of KDM2A by more than 3-fold after 4 h in SH-SY5Y cells (Fig 4D). In addition, propofol enhanced the expression of DNMT 3B in SH-SY5Y cells after 2h but recovered after 24h (Fig 4E). Similar effects could be detected in PBMCs (data not shown). The expression of DNMT1 methyltransferase was not affected by propofol (data not shown).

## Discussion

The use of propofol in anaesthesia is statistically associated with postoperative delirium (POD) [8, 9]. Since a causal relationship is currently unknown, we conducted this study to evaluate a possible epigenetic mechanism of how this anaesthetic drug could contribute to POD. We focussed our efforts on cholinergic genes, since a reduction in cholinesterase activity is discussed as a major factor for POD [15].

First, we tested the methylation of promoter regions. Of the three regions we evaluated (*ACHE*, *BCHE* and *CHRNA7*), only the methylation of the promoter region of *CHRNA7* was changed by propofol. Strikingly, by using ADC we could show that an artificial reduction of the methylation [25] of the promoter region of *CHRNA7* did not increase its expression but significantly reduced it. Thus, it seems appropriate to suggest that the region of the *CHRNA7* gene we investigated has suppressive effects on the transcription of this gene. Given that the basal methylation at that CpG was only about 6%, it is intriguing to speculate that the expression of *CHRNA7* could be much stronger impacted by an increase of the methylation of this region. Unfortunately, investigating this possibility was beyond the scope of this study and will be the subject of further investigations.

Secondly, since activation of Chrna7 can lead to suppression of cytokine release [23], we wondered whether a methylation of this repressor region (and subsequent downregulation of the gene) might increase cytokine release, further linking neuro inflammation to propofol treatment. While they tended to be upregulated after 24h, there was no significant effect of propofol on the expression of pro inflammatory secreted proteins such as TNFα or IL-6. This might indicate that the reduced activity of cholinesterases balances the downregulation of Chrna7 by increasing cholinergic signalling. Neither the expression of the anti-inflammatory cytokine IL-10 did in–or decrease significantly upon propofol treatment. Therefore, we can conclude, that propofol does not strongly contribute to pro–or anti–inflammatory signalling, both being discussed as additional factors in the development of POD [16–18].

Third: While propofol did not impact the methylation or the concentration of AChE or BChE, it did significantly reduce the activity of these proteins. This provides a mode of action of how propofol might induce, or contribute to POD. It is noteworthy that, while propofol reduced the activity of cholinergic enzymes, these effects on the activity of AChE / BChE were only transient and the activity levels were mostly restored after 24 h. This could be explained by the instability of propofol in cell culture dishes [26] and the high proliferation rate of SH-SY5Y cells, contributing to these cells being an accelerated model system. We are aware of the limitations that this model being a cancer cell line pose. However, we chose the neuronal cell line SH-SY5Y, because extraction of neuronal cells from patients with neurologic symptoms matching POD is not feasible [27]. Furthermore, SH-SY5Y cells depict an established cell line used to study brain disorders such as Parkinson or Alzheimer’s disease [28, 29]. Therefore, keeping the limitations of immortalized cell lines in mind, we feel confident that it is appropriate to perform our investigations in our selected cell line.

Fourth: An interesting observation is that the decreased activity of AChE / BChE is not rooted in an altered protein expression. While BChE concentration dropped slightly at 2h, it did not reach significant levels, and AChE concentration was unaffected by propofol. The slight drop of BChE concentration at 2h is interesting, nonetheless. Given that the half time of BChE is about 10 to 14 days [30], we can speculate that this effect is far too early to be a change in expression (which is also in line with the qPCR results) but could be due to an increased degradation. Further work is needed to fully investigate this phenomenon.

Fifth: If their expression is not changed, what does impact the activity of the cholinesterases? A shift in splicing could explain a reduced activity, while protein levels over all splice variants stay constant if one of the splice variants exhibited a lower activity. For AChE three major splice variants are listed at NCBI. *ACHE-S*, *ACHE-E* and *ACHE-isoform 4*. A fourth isoform (*ACHE-R*) is also reported but is usually only detected during stress [31]. We could detect the expression of 2 of the major splice variants *ACHE-S* and *ACHE-isoform 4*. The mRNA expression of these two transcripts both were upregulated over 24h (with the isoform 4 being statistically significant), possibly as a reaction to the decreased AChE / BChE activity. The *ACHE-isoform 4* codes for a shorter protein of only 525 aa. It is missing the amino acid residues from 357–444 of the AChE-S variant. The observed increase in *ACHE-S* mRNA levels in connection to a drop in activity levels of cholinesterases is in accordance with the work from Shaltiel et al [32] who could show that during stress AChE activity was down regulated in the brain, while the mRNA levels of *ACHE-S* were upregulated. They did not quantify the expression of the *ACHE-isoform 4*. To our knowledge nothing has been reported regarding the expression of AChE-isoform 4 in POD or related diseases. However, since we could not observe a change in the relevant time frame (2h – 4h) in either *ACHE-S* or *ACHE-isoform 4*, a splice variant specific expression change of these two splice variants can also be ruled out as reason for the reduced activity of AChE / BChE.

Sixth: Since propofol changed the activity of cholinergic proteins without changing their expression or their methylation profile, we tested whether propofol would change the epigenome of the cell on a broader level. This might impact cholinergic activity by changing the expression of an antagonist of these enzymes or by influencing post translational modifications of AChE or BChE [33]. Therefore, we investigated histone methylations and could find that the trimethylation of lysine 27 on histone 3 was significantly reduced upon propofol treatment. Tri-methylation of H3 K27 is widely associated with gene silencing by packing the chromatin tighter. It is especially well known for its role in X-chromosome inactivation [34]. A reduction of the tri-methylation of H3 K27 would therefore mean an overall increase in expression. Tri-methylation of H3 K27 as well as its methyltransferase EZH2 are connected to neuropathic pain in mice resulting in a change of the expression of a range of different genes [35]. Since changes in the methylation of histone 3 are in line with our theory of a larger–unspecific–change of the epigenome, we tested the expression a set of different DNA and histone modifying enzymes in our cell line, and because POD is also associated with an altered cholinesterase activity in blood samples [20], we investigated the expression of these enzymes in our cells line as well as PBMCs. Of these DMNT3B, KDM2A and HDAC1 showed visible changes in expression after propofol treatment in both SH-SY5Y cells and PBMCs, potentially rewiring the epigenetic landscape of the cell. In mice Sailaja et al could show that stress induced a similar upregulation of epigenome maintenance genes (such as HDAC1) which coincided with a significantly reduced expression of AChE-S [36]. They speculated that there might be a causal link between these histone modifying genes and *ACHE-S* expression. However we cannot observe such an effect, since *ACHE-S* expression tends to be strengthened in coincidence with HDAC1 expression in our cells. Further work is needed sequencing the complete epigenome of the cell and utilizing gene expression profiles in order to better understand the extensive changes propofol might induce in the cells. Furthermore, epigenetic modifications have already been observed for the use of a different anaesthetic drug, sevoflurane [7], even if the authors did not establish a connection to POD. This hints at the fact, that widespread re-wiring of the epigenome might be a common result of anaesthesia and should be more systematically investigated in further studies.

We can only speculate if the epigenetic effects influence this reduction of choline esterase activity or whether they contribute to POD in a different way or at all.

## Conclusions

In summary, we found that the anaesthetic drug propofol reduces the cholinergic activity, suggesting a mode of action for association of propofol anaesthesia with post-operative delirium. Furthermore, propofol treatment changed the expression of DNA and histone modifying genes on a global scale. We can speculate that this might result in a wide spread re-wiring of the epigenome in propofol treated neuronal cells and possibly impact cholinergic activity by influencing the post translational modifications of these proteins. We call for a systematic investigation of how and to which extent anaesthetics change the epigenome of cells and have potentially an effect far exceeding the time of anaesthesia.

## Abbreviations

AChE: acetylcholinesterase protein
ACHE: acetylcholinesterase gene
ACTB: actin beta gene
AS: antisense
BChE: butyrylcholinesterase protein
BCHE: butyrylcholinesterase gene
cDNA: complementary DNA
ChIP Assay: chromatin immunoprecipitation assay
CHRNA7: nicotinic acetylcholine receptor 7 gene
Chrna7: nicotinic acetylcholine receptor 7 protein
CO2: carbon dioxide
DMEM: Dulbecco’s Modified Eagle Medium
DNA: deoxyribonucleic acid
DNMT: DNA methyltransferase
DNMT 1: DNA methyltransferase 1
DNMT 3B: DNA methyltransferase 3B
EDTA: ethylenediaminetetraacetic acid
ELISA: enzyme-linked immunosorbant assay
FCS: foetal calf serum
Fig: figure
H; K: histone; lysine
PBMC: peripheral blood mononuclear cells
PBS: phosphate buffered saline
PCR: polymerase chain reaction
Penstrep: penicillin/streptomycin
PMSF: phenylmethylsulfonylfluorid
POD: postoperative delirium
Propofol: 2,6-Diisopropylphenol (Sigma-Aldrich, Taufkirchen, Germany
RNA: ribonucleic acid
RPMI: Roswell Park Memorial Institute medium
SE: sense
KDM2A: lysine demethylase 2A
TEB-buffer: triton extraction buffer
TNFα: tumour necrosis factor alpha
